# Topology-controlled self-assembly of amphiphilic block copolymers†

**DOI:** 10.1039/d3nr01204b

**Published:** 2023-08-30

**Authors:** Raquel López-Ríos de Castro, Robert M. Ziolek, Christian D. Lorenz

**Affiliations:** a Biological Physics and Soft Matter Group, Department of Physics, King's College London London WC2R 2LS UK chris.lorenz@kcl.ac.uk +44 207 848 2639; b Department of Chemistry, King's College London London SE1 1DB UK

## Abstract

Contemporary synthetic chemistry approaches can be used to yield a range of distinct polymer topologies with precise control. The topology of a polymer strongly influences its self-assembly into complex nanostructures however a clear mechanistic understanding of the relationship between polymer topology and self-assembly has not yet been developed. In this work, we use atomistic molecular dynamics simulations to provide a nanoscale picture of the self-assembly of three poly(ethylene oxide)-poly(methyl acrylate) block copolymers with different topologies into micelles. We find that the topology affects the ability of the micelle to form a compact hydrophobic core, which directly affects its stability. Also, we apply unsupervised machine learning techniques to show that the topology of a polymer affects its ability to take a conformation in response to the local environment within the micelles. This work provides foundations for the rational design of polymer nanostructures based on their underlying topology.

## Abstract 2

Our molecular dynamics simulations provide molecular-scale understanding of how polymer topology effects the self-assembly and stability of nanoparticles, and the polymer molecule’s ability to take a conformation in response to its local environment.

## Graphical abstract

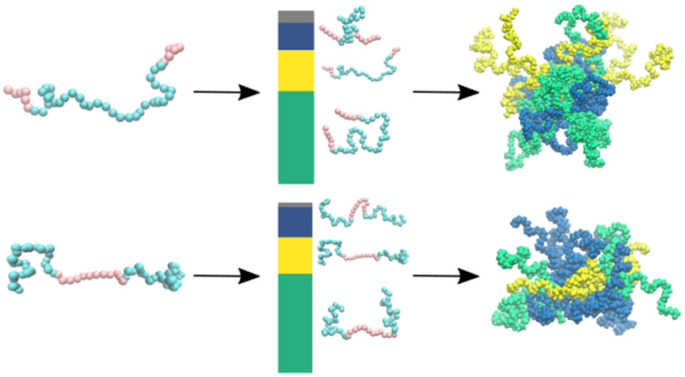

## Introduction

The ability of amphiphilic polymers to self-assemble into specific morphologies in solution has driven interest in their deployment for a diverse range of applications.^1–4^ The topology of block copolymers exerts great influence over their properties and therefore their potential applications. Ring polymers are one synthetically accessible topology that have drawn considerable attention as a result of the unique properties that they exhibit in comparison to their linear counterparts.^5–13^ Functional polymer nanostructures have been typically fabricated using linear polymers but significant synthetic advances in the past two decades have made ring copolymer synthesis possible. Ring polymers demonstrate distinct self-assembly behavior,^9,12,13^ which leads to their resultant micelles possessing markedly different properties,^9^ including the size and shape,^14^ morphology,^15,16^ temperature, salt tolerance,^17,18^ and degradation^14^ with respect to micelles formed from analogous linear polymers.

In drug delivery applications, the ability to control the size and stability of micellar aggregates is particularly important. The size of such micelles is one of the most critical features in determining biodistribution and the stability can be tuned to prevent premature release or to enable a controlled release of therapeutics. Ring polymers have shown great promise as potential drug and gene delivery vehicles because they often show improved drug loading and releasing capacity,^19,20^ greater efficacy,^21–24^ longer *in vivo* circulation times,^25,26^ and high cancer cell uptake^25–28^ as the same polymers with a linear topology.

While interest in the application of self-assembling ring polymers in drug-delivery applications is building, there is a relative lack of detailed understanding of the molecular-scale mechanisms that drive the emergence of their desirable properties. Molecular-scale simulations present the unique opportunity to build this level of understanding. Simulations have recently been used to develop understanding of the unique properties of ring polymers within polymeric melts,^29,30^ extensional flows^31,32^ and thin films.^33,34^ However, relatively few simulation studies have investigated the underlying mechanisms that lead to the properties of ring polymers in aqueous environments observed experimentally. Studies that have been performed have primarily utilized coarse-grain polymer models to gain insight into how polymer topology affects the morphology of the micelles that form.^23,35–39^

**Fig. 1:**
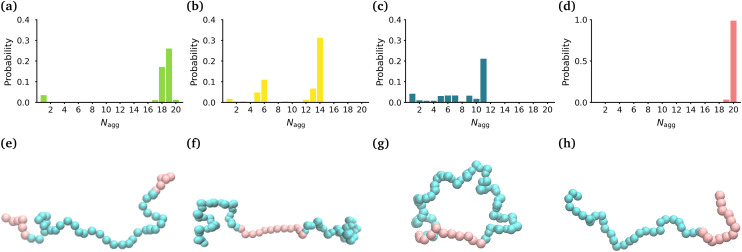
Size and shape of micelles. Probability distribution of *N*_agg_ for the (a) MA-terminated polymers, (b) EO-terminated polymers, (c) ring polymers and (d) diblock polymers. Snapshots of the (e) MA-terminated (f) EO-terminated, (g) ring polymers and (h) diblock polymers. MA is shown in pink and EO in blue.

In this manuscript, we employ all-atom molecular dynamics simulations to gain a detailed understanding of the atomistic interactions and molecular mechanisms that drive the self-assembly of a ring polymer consisting of poly(methyl acrylate) and poly(ethylene oxide) blocks (-(MA_12_EO_31_-)) in comparison to its analogous linear diblock topology (MA_12_EO_30_) and triblock topologies (MA-terminated (MA_6_EO_31_MA_6_) & EO-terminated (EO_15_MA_12_EO_15_)) (see Fig. 1(e)–(h)). We provide a detailed description of the internal structure of the micelles that each polymer forms, which plays a key role in drug solubilization, as well as the stability of micelles as drug delivery vehicles.

## Results

### Effect of polymer topology on the size & shape of micelles

In order to determine the size, shape and compactness of the micelles formed by the different polymers, we measured the number of polymer molecules in each micelle within our simulations at stationarity. We also measured the radius of gyration (*R*_G_) and the eccentricity of the largest micelle in each system (Fig. S2†).

Fig. 1(a)–(d) shows the probability distribution of the aggregation number for the different topologies. The MA-terminated linear polymers form one micelle which contains approximately 19 (of the 20) polymers (Fig. 1(a)). The EO-terminated linear polymers self-assemble into two micelles, one with approximately 14 polymers and the other with 6 polymers (Fig. 1(b)). The ring polymers form multiple micelles with the largest one containing approximately 11 polymers (Fig. 1(c)). Finally, the diblock polymers predominantly form one micelle with all 20 polymers (Fig. 1(d)). The values of *R*_G_ correlate directly with the aggregation numbers, such that the diblock polymer micelle has the largest *R*_G_, followed closely by the MA-terminated linear polymer one and then, in decreasing order, the EO-terminated linear polymer and the ring polymer (Table 1). Despite the difference in size of the micelles for the four different polymers, all of the micelles are approximately spherical (eccentricities ∼0.1) (Table 1).

**Table 1:** Effect of polymer topology on the size and shape of micelles. The average and standard deviation for the *R*_G_, the eccentricity *ε* and the average aggregation number *N*_agg_

Topology	*R*_G_ (Å)	*ε*	*N*_agg_
MA-terminated linear	28.2 ± 1.4	0.10 ± 0.06	19 ± 1
EO-terminated linear	23.3 ± 0.5	0.09 ± 0.06	14 ± 1
Ring	19.5 ± 0.4	0.07 ± 0.04	11 ± 1
Diblock	29.3 ± 0.9	0.10 ± 0.07	20 ± 1

We have also carried out simulations of each of the different polymer topologies that contain 30 polymers at the same concentration as in the 20 polymer simulations. We found the very similar aggregation numbers in these larger systems for each of the topologies, except for the diblock (see Fig. S3†). In the diblock system, we once again see that nearly all of the polymers self-assemble into a single micelle.

### Effect of polymer topology on the internal structure of micelles

**Fig. 2:**
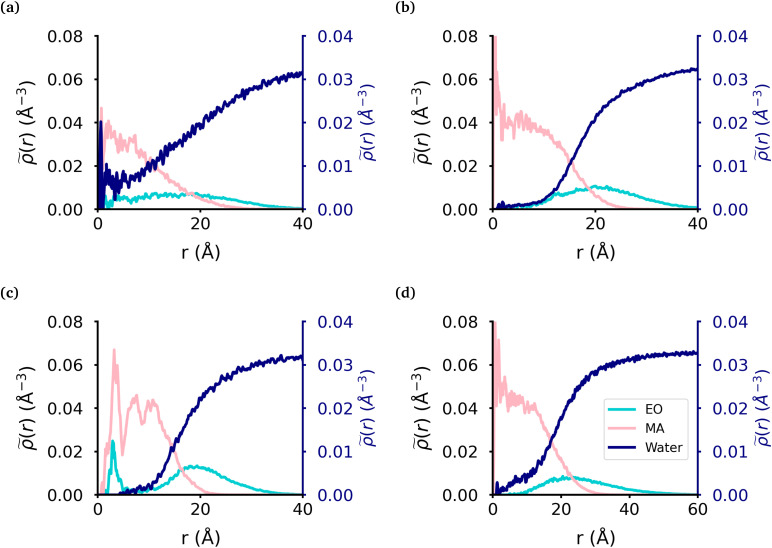
Radial density of micelle components. The radial density of micelles formed from the (a) MA-terminated polymer, (b) EO-terminated polymer, (c) ring polymer and (d) diblock polymer. MA monomers are displayed in pink, EO in blue and water in dark blue.

We calculated the radial density (Fig. 2) of the micelles, as well as the corresponding intrinsic density using the intrinsic core–shell interface (ICSI) method^40^ (Fig. S6†), in order to understand how the internal structure of each micelle is affected by the topology of each polymer. For all topologies, the corona of the micelle is constituted primarily of the EO blocks. In the case of the MA-terminated linear polymer, we observe that approximately 20% of the polymers have at least one MA-terminated end in the corona of the micelle. Therefore, the micelle core formed by these polymers has significantly more EO monomers and as a result, more water, present in its core than either of the other micelles (Table 2). Regarding the other topologies, the diblock, EO-terminated linear polymer and the ring polymer have a small amount of EO monomers in the core (Fig. 2(b)–(d) & Table 2). However the ring polymer has a slight increase in the density of EO monomers (also seen in the intrinsic densities as shown in Fig. S8†) in the core as there are no free ends of the polymer, instead both ends of the EO block are attached to MA blocks. As there is a peak in the MA density which corresponds to the peak in EO density in the core of the micelle, it is clear that the peak in the EO density is a result of its connectivity to the MA monomers. It should also be noted that while the peaks in each curve look significant, as the volume measured that close to the core of the micelle is quite small and so the actual amount of EO is quite small.

**Table 2:** Hydration of the micelle core

Topology	MA_H_2_O_	EO_H_2_O_	EO_core_
MA-terminated linear	3.0 ± 1.0	10.6 ± 3.8	51.0 ± 15.8
EO-terminated linear	0.4 ± 0.2	4.6 ± 3.1	7.9 ± 3.2
Ring	0.5 ± 0.1	2.3 ± 0.9	11.7 ± 3.0
Diblock	0.5 ± 0.1	8.8 ± 6.5	11.7 ± 6.4

**Fig. 3:**
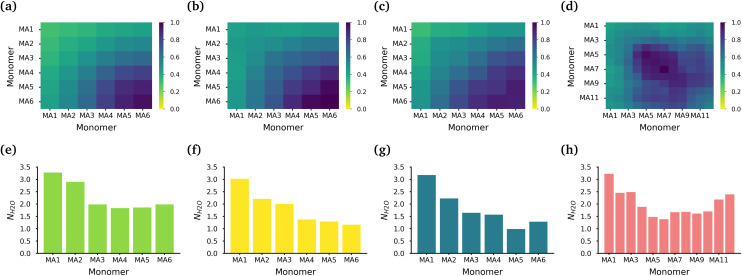
Interactions within the MA core of the polymer micelles. The normalized intermolecular MA contacts within the core of the (a) MA-terminated polymer, (b) EO-terminated polymer, (c) ring polymer and (d) diblock polymer micelles. Average hydration of the carbonyl oxygen atoms in the PMA backbone of the (e) MA-terminated polymers, (f) EO-terminated polymers, (g) ring polymers and (h) diblock polymers.

The core of each micelle consists primarily of MA blocks. Fig. 3(a)–(d) show the normalized intermolecular contacts of the (chemically equivalent, except for the case of the diblock, where there are no chemically equivalent atoms) MA monomers in the MA-terminated linear, EO-terminated linear, ring and diblock polymer micelles. In all topologies except the diblock polymer, the number of contacts increases the further a MA monomer is from the EO blocks in each polymer with MA6, the monomer furthest away from the EO blocks, undertaking the highest number of contacts. In the diblock polymer micelles, the MA monomers that are closest to the EO blocks, are also the monomers with the lowest number of contacts. But in this case, the MA monomers found in the middle of the PMA block are the ones that have the highest contacts.

While all of the MA monomers contribute to the micelle's core, the MA monomers furthest away from the EO blocks are the monomers that play the most significant role in the formation and stabilization of the micelle core. The MA-terminated linear polymers have the lowest number of contacts between their MA monomers as a result of the MA monomers being divided into two blocks which are separated by the block of EO monomers and the number of MA monomers outside the core. Fig. 3(e)–(h) show the normalized number of water molecules within the first hydration shell of the carbonyl oxygens in the different chemically equivalent MA monomers within each polymer. The MA-terminated linear polymers have the largest coordination number values, which is consistent with the measured water densities that demonstrate that more water is found within the core of this micelle. In all micelles, the most hydrated monomer is MA1 which is directly bonded to an EO monomer, and generally the hydration decreases as the monomer is further from the EO monomers.

### Effect of polymer topology on polymer conformations within micelles

**Fig. 4:**
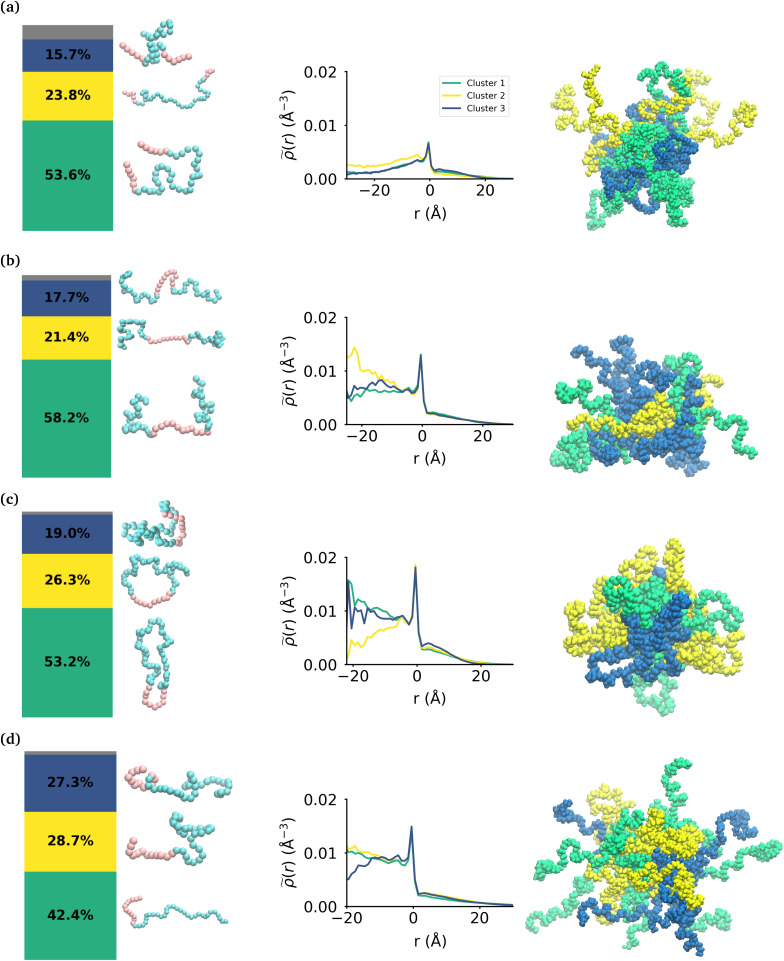
Effect of topology on the internal structure of the polymer micelles. From left to right, a bar chart shows the percentage of each cluster of conformations within the micelle, then there are representative snapshots of the polymers within each cluster, and then plots of the intrinsic density of the various clusters within the micelle and finally a snapshot of the micelle with each polymer color-coded for the cluster it belongs to. These are shown for the (a) MA-terminated polymer micelle, (b) EO-terminated polymer micelle (c) ring polymer micelle and (d) diblock polymer micelle. Sizes are not to scale.

While the MA monomers are key in the formation and stability of the micelles, the conformations that each topology of the polymers take within the micelle is significantly different. To investigate the specific conformations that different polymers adopt within a micelle, we applied a two step machine learning protocol:^41^ dimensionality reduction using the Uniform Manifold Approximation and Projection (UMAP) algorithm,^42^ followed by clustering in the resulting embedded space using Hierarchical Density-Based Spatial Clustering of Applications with Noise (HDBSCAN)^43^ (see the ESI section:† ‘Dimensionality reduction and clustering’ for the full methodology and results of this protocol). In each embedding, three clusters were identified representing the different groupings of similar conformations taken by each polymer (see Fig. S7†). In each case, there is less than 8% of the data that is not clustered by HDBSCAN, which is shown in the bar charts in gray. Fig. 4 shows the probability distribution of each cluster in the various micelles as well as representative structures of each cluster of conformations for each polymer. The representative structures show that the conformations are clearly differentiable by the relative extension of the EO and MA blocks.

We then use the ICSI method to measure the location of the various polymer conformations within each micelle (Fig. 4). Snapshots of each micelle with its constituent polymers colored by the corresponding cluster number are also shown in Fig. 4. In the MA-terminated linear polymers, the intrinsic densities of the various clusters are less than found in the other micelles, which is indicative of more water present in the core as shown in Fig. 2(a). Also there is not a significant difference in the distributions of the three conformations within the micelle. The most extended conformation (cluster 2) is representative of the previously mentioned polymers that have at most one MA block in the core of the micelle, and is also slightly more commonly found in the core. In the EO-terminated linear and cyclic micelles that have a more stable core, the polymers take specific conformations depending on their position within the core of the micelle. For example, the most extended conformation of the EO-terminated linear polymers (cluster 2) is more likely to be found in the core of the micelle with the MA block spanning the micelle and the two EO blocks extended into solution. Closer to the core–shell interface, there is an increased density of the other two conformations which have more collapsed MA blocks resulting in the MA monomers shielding the core of the micelle from the surrounding water.

For the ring polymer micelle, the adopted conformations that are most elongated (clusters 1 and 3) are found to be enriched in the core of the micelle. In these conformations, the EO block is more extended so that it can reach the micelle corona and interact with the surrounding aqueous environment. The polymers at the interface of the core of the ring polymer micelle take on a more conventional ring shape (cluster 2), allowing the EO block to expand to maximize its contact with the surrounding water and the MA block to embed into the core to minimize its interaction with water.

For the diblock polymer micelle, the pattern is similar as for the cyclic one. The most extended conformations (cluster 1 and 2) are predominate in the core of the micelle. In these conformations, the MA is more extended, allowing it to maximize its contacts with the rest of the MA present in the core. Finally, cluster 3 is more likely to be found at the core–shell interface. This cluster presents a collapsed MA and extended EO, which allows the EO to maximise its contacts with the water, while the MA minimizes its contacts with this solvent by collapsing within itself.

## Discussion

The results of our simulations show excellent agreement with previous experimental work studying the effect of topology on the self-assembly of block copolymers. In this work, we show that the linear polymer with the hydrophobic monomers on either end of the polymer (MA-terminated linear polymer) forms larger aggregates that are less stable than those formed from the cyclic or diblock polymer. Honda *et al.* have studied MA-EO-MA linear and MA-EO ring block copolymers and found that the linear polymers form micelles that have larger hydrodynamic diameters and aggregation numbers, while also being less thermally and salt stable than the corresponding ring polymer.^18^ The same authors also studied butyl acrylate (BA)-ethylene oxide linear and cyclic block copolymers and found that the size of the micelles from the two polymers were similar but the ring polymer showed greater thermal stability.^17^ Our simulations show that there are more MA–MA contacts within the core of ring polymer as compared to the MA-terminated linear polymer which results in a more compact (ring: ∼119 Å^2^ per polymer; MA-terminated linear: ∼124 Å^2^ per polymer) and more stable micelle (ring has smaller fluctuations in *R*_G_ than MA-terminated; Table 1). We also find that the MA-terminated linear polymers form micelles which have a significant number of EO monomers internalized into the core of the micelle which results in there being a significant amount of water within the core (Table 2). This increased amount of the water in the core reduces the stability of the micelle (Table 1).

Our ability to identify three distinct conformations of each of the polymers allows us to provide a detailed picture of the internal structure of the micelles. In doing so, we show that for the linear polymer with the hydrophobic monomers on either end (MA-terminated linear) there are two conformations where the MA blocks are near to one another and one conformation in which the polymer is fully extended with the MA blocks separated from another. This is consistent with the general picture suggested for the MA-EO and BA-EO polymers studied by Honda *et al.*^17,18^ as well as for Pluronics which contain blocks of propylene oxide (PO) and ethylene oxide.^44^ In each case, the authors suggest that these polymers with the hydrophobic monomers on the terminal ends form flower-like micelles where a majority of the polymers have both terminal ends within the core of the micelle, and some of the polymers have a hydrophobic terminal end in solution. The results of our simulations for the MA-terminated linear polymers show that ∼20% of the polymers take conformations which result in at least one of the MA-blocks being in the corona of the micelle. Interestingly, with the larger aggregation number for the MA-terminated linear polymers than for the micelles formed from the EO-terminated linear polymers, we find that both micelles have roughly the same number of MA monomers (∼360) in the core of their micelles.

We found that in the micelles formed by the EO-terminated triblock, the diblock and the ring polymers, which have a well defined core and corona, the polymers take different conformations depending on their location within the micelle. In the case of the EO-terminated linear polymer we find that the polymers in the core of the micelle have a propensity to have an elongated MA block which maximizes the hydrophobic contact between MA monomers and more compact EO blocks which lie on the surface of the micelle. The polymers at the core/shell interface of the micelle have more compact MA blocks which allows the polymers to more effectively shield their hydrophobic blocks and the EO blocks are more extended in order to maximize their hydration. While in the ring polymer micelle, we find two more elongated conformations which are most prominent in the core of the micelle, whereas the other more ring-like conformation sits at the core–corona interface. These conformations taken by the ring polymers in the different parts of the micelles allow the polymers to maximize the hydrophobic contact of the MA blocks while also allowing the EO monomers to maximize their interaction with the surrounding water. In the case of the diblock polymer micelle, we find that the conformations where the MA blocks are the most extended are located closer to the core, while the conformation with a collapsed MA block is found close to the core–shell interface. Then, it is clear that these conformations are the result of the MA monomers maximising their hydrophobic interactions and minimising their contact with the aqueous environment. Therefore our findings show that polymers that can take location specific conformations will form stable micelles that have hydrophobic cores which are shielded by the hydrophilic monomers, and those that cannot, the MA-terminated polymer in this case, will not.

## Conclusions

Our simulations provide a mechanistic picture of what leads to the difference in size and stability of micelles formed by block copolymers that differ in topology but not in the chemical composition of their constituent monomers. Additionally, we have been able to demonstrate the range of conformations that are taken by four different topologies of polymers within the micelle and how they determine the stability of the micelles. We have also shown how the conformations of the polymers change as their position within the micelle changes, which is particularly interesting when considering loading these micelles with small molecule therapeutics, as the location and the hydration of the drug within the micelle will be driven largely by the conformations of the polymers in its local environment. This understanding allows polymer topology to become another parameter that can be used to perform rational design of polymer nanoparticles for the use in a variety of applications including drug delivery.^45–47^

## Methods

Each simulation reported consists of 20 polymers placed in a simulation box with initial dimensions of 147 Å × 147 Å × 147 Å containing approximately 105 000 water molecules, resulting in 3 wt% solutions of each polymer. We used the OPLS forcefield parameters as prescribed by the PolyParGen webserver^48^ to describe the interactions of the polymers and the TIP3P water model.^49^ All of the simulations were performed using GROMACS^50^ versions 2019.2 and 2020.4. The same simulation protocol was followed for each of three simulations, which begins with energy minimization by steepest descent, followed by a 125 ps simulation in the NVT ensemble using the Nosè–Hoover thermostat to control the temperature (target temperature 300 K) with a timestep of 1 fs. Subsequently we ran 1 μs production simulations in the NPT ensemble using the Nosè–Hoover thermostat and the Parrinello–Rahman barostat to control the temperature (target temperature 300 K) and pressure of 1 atm, respectively with a 2 fs timestep while all hydrogen-containing bonds were constrained using the LINCS algorithm.^51^ In all simulations, the non-bonded interactions were cut off at 12 Å while the particle-mesh Ewald (PME) algorithm was used to calculate long-range electrostatic interactions. Appropriate burn-in times were calculated, with only the stationary portion of the production simulations used for analysis. A description of all of the analyses conducted on these simulations is described in the ESI.†

## Author contributions

Raquel López-Ríos de Castro: data curation, formal analysis, investigation, methodology, software, validation, visualisation, writing – original draft. Robert M. Ziolek: conceptualization, formal analysis, methodology, software, supervision, writing – review & editing. Christian D. Lorenz: conceptualization, funding acquisition, project administration, resources, supervision, writing – review & editing.

## Conflicts of interest

There are no conflicts to declare.

## Supplementary Material

NR-015-D3NR01204B-s001
