# Supplementary material for: Topology-controlled self-assembly of amphiphilic block copolymers
Source: Nanoscale. 2023 Aug 30;15(37):15230–7. doi: 10.1039/d3nr01204b (PMC10540979; doi:10.1039/d3nr01204b)
Supplement: NR-015-D3NR01204B-s001 [file NR-015-D3NR01204B-s001.pdf]

Electronic Supplementary Material (ESI) for Nanoscale.
This journal is © The Royal Society of Chemistry 2023

Supplementary Information for "Topology-Controlled Self-Assembly of Amphiphilic Block Copolymers"

Raquel López-Ríos de Castro,[†,‡] Robert M. Ziolek,[‡] and Christian D. Lorenz[*,‡]

†*Department of Chemistry, King's College London, London, SE1 1DB, United Kingdom*

‡*Biological Physics and Soft Matter Group, Department of Physics, King's College London, London, WC2R 2LS, United Kingdom*

E-mail: chris.lorenz@kcl.ac.uk

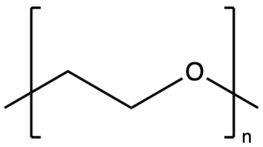

(a) PEO

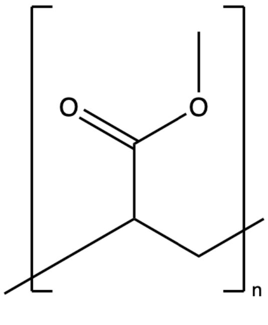

(b) PMA

Figure S1: (a) Molecular structure of PEO (b) Molecular structure of PMA.

Analysis of simulations All analysis was carried out with Python 3.9.5. The Python modules MDAnalysis,[1] pySoftWhere[2] and NetworkX,[3] the algorithms of UMAP[4] and HDBSCAN[5] were used to develop in-house scripts for this analysis.

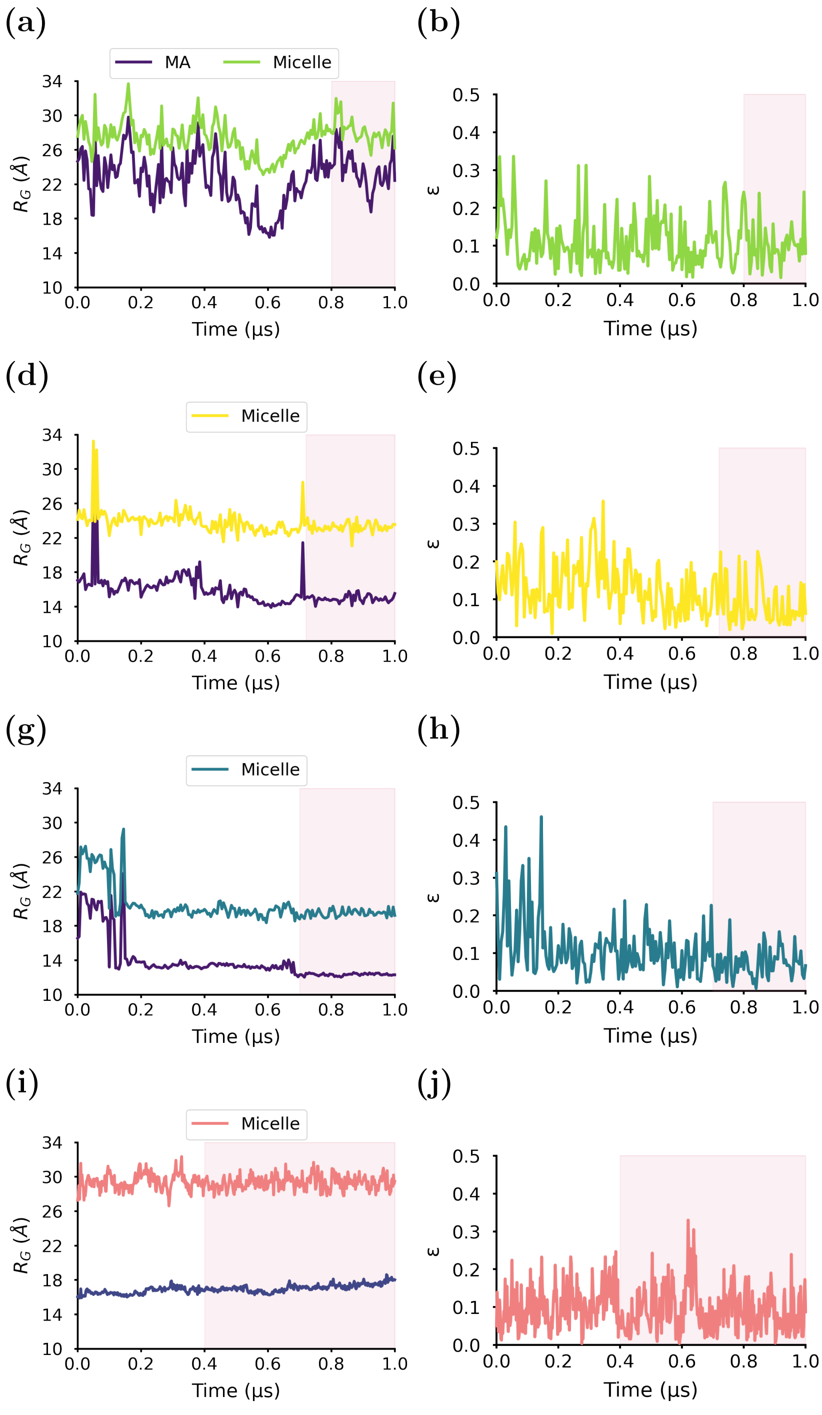

Figure S2: **Size and shape of micelles.** Plots of the R_{G} for the largest micelle as a function of time for the (a) MA-terminated polymer micelle, (d) EO-terminated polymer micelle, (g) ring polymer micelle and (i) diblock polymer micelle. Plots of the eccentricy of the largest micelle as a function of time for the (b) MA-terminated polymer micelle, (e) EO-terminated polymer micelle, (h) ring polymer micelle and (j) diblock polymer micelle. The time scale over which each micelle has reached equilibrium is shaded in pink.

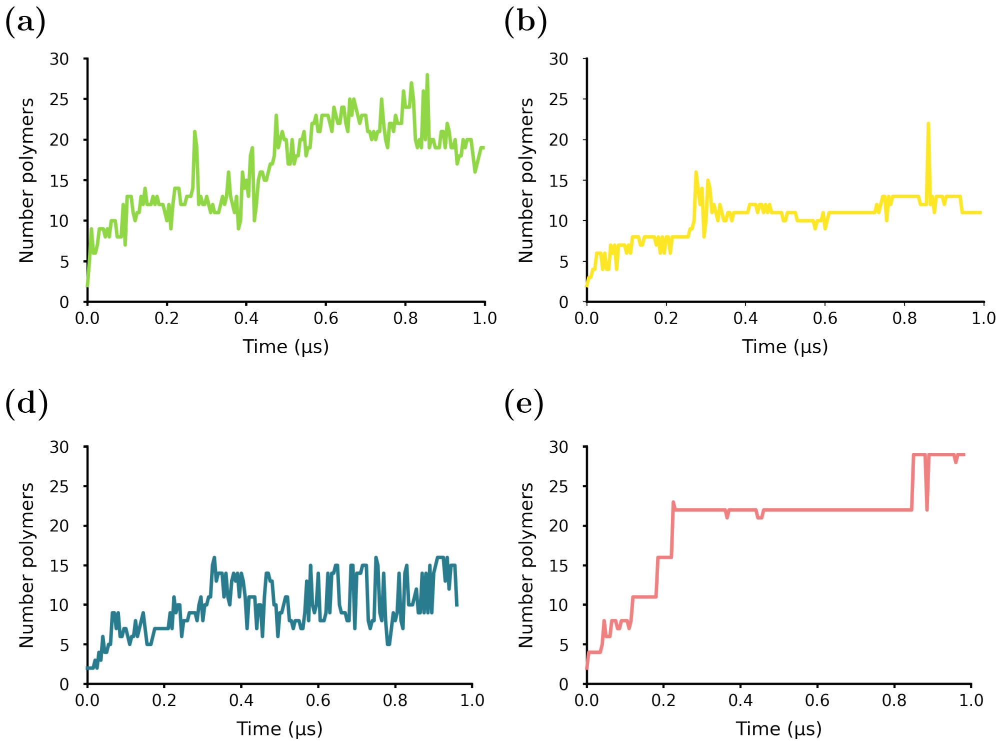

Figure S3: **Largest micelle size in 30 polymer simulation.** Plots of the number of polymers belonging to the biggest micelle over time for a) triblock ma terminated, b) triblock eo terminated, c) cyclic and d) diblock polymer topologies, for simulations with 30 polymers but same water to polymer ratio as for the simulations of 20 papers tated everywhere else in the manuscript. This proves that there is no system size effect for these systems and in fact we would expect if you had even more polymers the largest micelle would contain the same number of polymers but there would be multiple of the same size. Except for the case of the EO-MA diblock polymer (simulated in order to address a question raised by Reviewer 1), wherecase it seems that those polymers phase-separate instead of form micelles, and therefore their micelle size continues to increase

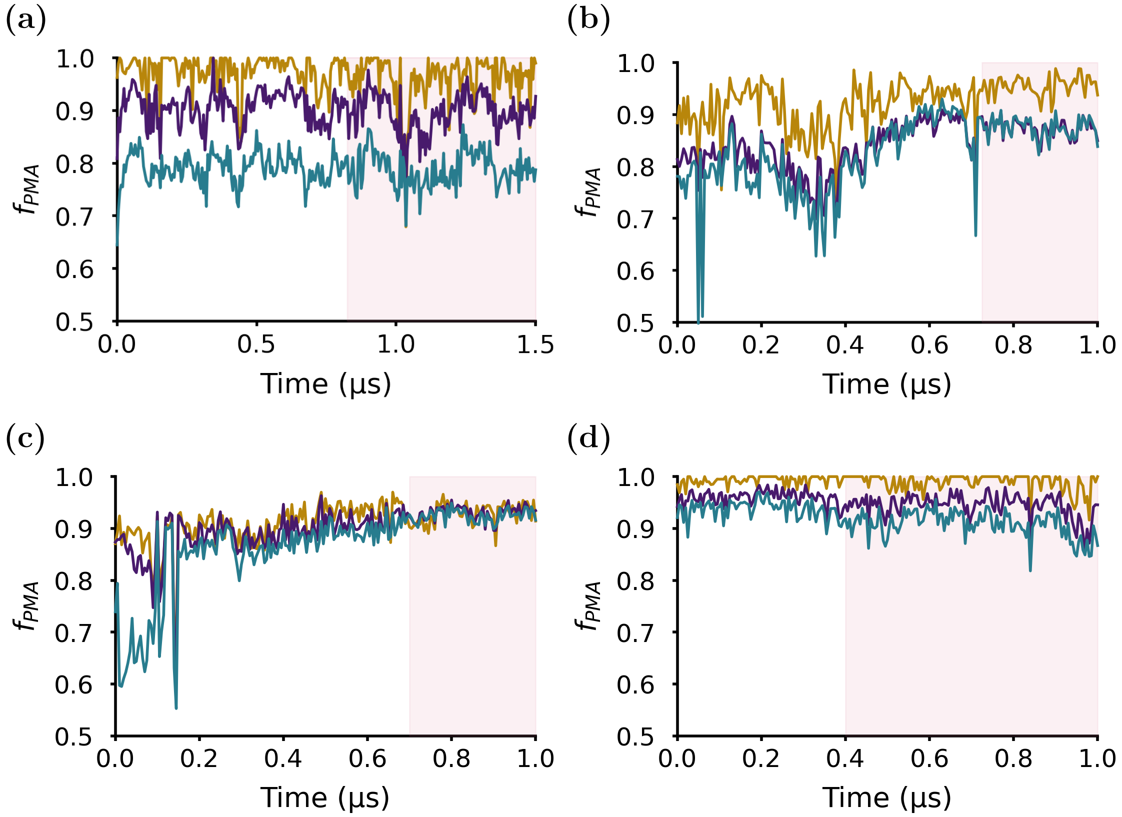

Figure S4: **Equilibration analysis** Fraction of PMA heavy atoms that are in the core with respect to all heavy atoms of the polymers species in the core (a) MA-terminated polymer micelle, (b) EO-terminated polymer micelle and (c)ring polymer micelle. The brown line represents the fraction of PMA atoms at a distance within 10$\mathring{A}$ from the core, the purple line is within a distance of 15$\mathring{A}$ and the blue line is a distance below the radius of gyration of the core.

The R_{G} of the micelle, is described by Equation (1)

$$R_{\mathrm{G}} = \sqrt{\frac{1}{M}\sum_{i=1}^{N} m_i (r_i - R)^2} \tag{1}$$

where M is the total mass of the body, m_i is the mass of atom i and R is the mean position of all atoms. Both, the R_G of the core of the NP and of the whole NP were calculated with the MDAnalysis function `radius_of_gyration()`.

In order to calculate the eccentricity (ϵ) of the whole and the core of the micelle, the moment of inertia around the principal axis were obtained with the MDAnalysis function

`moment_of_intertia()`. The eccentricity was obtained from Equation 2

$$\epsilon = 1 - \left(\frac{I_{min}}{I_{ave}} \right) \tag{2}$$

where I_{ave} is the average across all moments of inertia and I_{min} is the moment of inertia around the x axis.

Identifying the amount of polymers clustered in each micelle was done with the Python module Networkx, which is able to study the structure of complex NetworkX. Each polymer was defined by two atoms (triblock topologies) or one atom (ring topology), if polymers were closed enough via these atoms, they were considered part of the same network, which meant they formed part of the same micelle. The atom selection chosen in all topologies were MA atoms, as these form the hydrophobic core.

The number of contacts was performed using MDAnalysis tools. First, to define the intermolecular contact distance between the main carbon backbone atom of the different monomer species and water, we used the pair radial distribution function `rdf_calc.InterRDF` between the molecules that we wanted to study. The second peak in the rdf plots was used to determined the contact distance between the specific pair, which in this case was 7 Å. Afterwards, a contact was counted if the distance between the selected monomer atoms was below the assigned contact distance. For the hydration a similar procedure was followed, but the distances was selected to be approximately 4 Å and the water atom chosen for the contacts was the water oxygen.

When the contact results were plotted, the values were normalized by dividing the number of contacts between a pair of atoms by the number of configurations used in the analysis. Then, it was divided by the mean cluster size, to account for micelles being formed by different numbers of polymer molecules. To finalize the normalization across all simulations, the number of contacts between pairs of atoms was divided by the maximum number of contacts found across all three topologies.

Intrinsic Core-Shell Interface (ICSI) Method. For micelles with an irregular internal and interfacial structures, intrinsic interface techniques can be used to investigate the interfacial structure of micelles.[6] The intrinsic density was calculated with an intrinsic core-shell interface (ICSI) method provided by the python package pySoftWhere.[2] For this method we selected the MA heavy atoms to form the ICSI as they are the principal component of the core (this information could be inferred by the contacts maps, the hydration data and the spherical density of components). The grid selected was 30×30. Detailed information on the working of this algorithm can be found in (Ziolek et al).[6] The ICSI equation is:

$$\tilde{\rho}(r) \equiv \left\langle \sum_i \frac{\delta[r - (r_i - \xi(\theta, \phi))]}{\bar{S}_i(r)} \right\rangle \tag{3}$$

where r_i is the r-position of atom i (of the chosen group of atoms) and $\xi(\theta, \phi)$ is the r-position of the ICSI. The average volume of the shell in which a given atom is found when using the intrinsic surface approach, $\bar{S}_i(r)$, which normalizes the intrinsic density, is given by:

$$\bar{S}_i(r) = \frac{n_i \bar{V}_{\text{box}}}{N} \tag{4}$$

where n_i is the number of points found in the shell in which atom i is found over all the clusters analyzed, $\bar{V}_{\text{box}}$ is the average volume of the simulation box, and N is the total number of random coordinates used in the normalization procedure.

Dimensionality reduction and clustering. The distances chosen as the input space to generate the two dimensional UMAP embedded data are topology specific. The goal was to find the minimum number of distances that could resume the conformational complexity adopted by the polymers. First, for the MA-terminated polymer 5 distances were selected: distance between terminal MAs, both terminal MAs to central EO, and last EO monomers to central EO. Similarly, for the EO-terminated polymer, 5 distances were also selected. Distance between terminal EOs, distance between terminal EOs and central MA, and distance between the last MA monomers and the central MA. Finally, for the ring polymer only 4 were

needed. Middle MA to middle EO, terminal MA to middle EO and the distance between the last MA monomers. The UMAP embedded output was latter cluster with HDBSCAN, Table S1 shows the UMAP and HDBSCAN parameters chosen for each topology.

The intrinsic density of the UMAP clusters was calculated in the same as for the overall micelle intrinsic density. But instead of using all polymers, only the ones belonging to the specific cluster density being calculated were used.

Table S1: UMAP (*n_neighbours*) and HDBSCAN (*min_cluster_size* and *cluster_selection_epsilon*) parameters

	MA-terminated	EO-terminated	Ring	Diblock
n_neighbours	5	6	8	12
min_cluster_size	25	55	34	55
cluster_selection_epsilon	0.8	1	1.1	0.85

Table S2: Micelle equilibration time and the mean micelle size (Å).

	MA-terminated	EO-terminated	Ring	Diblock
Equilibration time (μs)	0.8	0.72	0.6	0.4
Mean micelle size	18.6 ± 0.6	13.7 ± 0.8	10.9 ± 0.4	20.0 ± 0.2

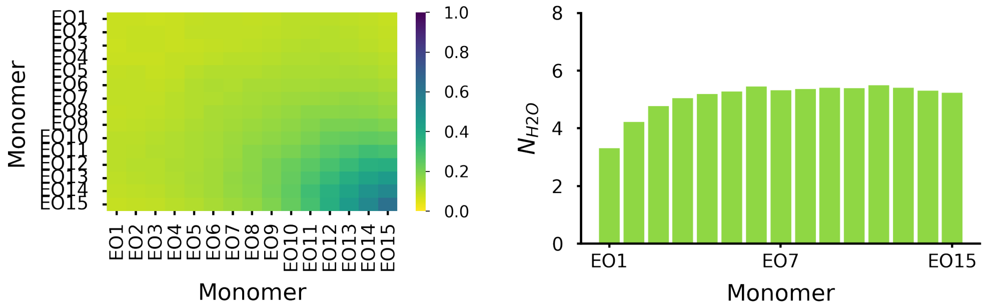

(a) EO-EO contacts (b) EO hydration

Figure S5: (a) Normalised intermolecular contact maps between the EO monomers and (b) normalised hydration of the oxygen atoms of the EO monomers for the MA-terminated polymer micelle.

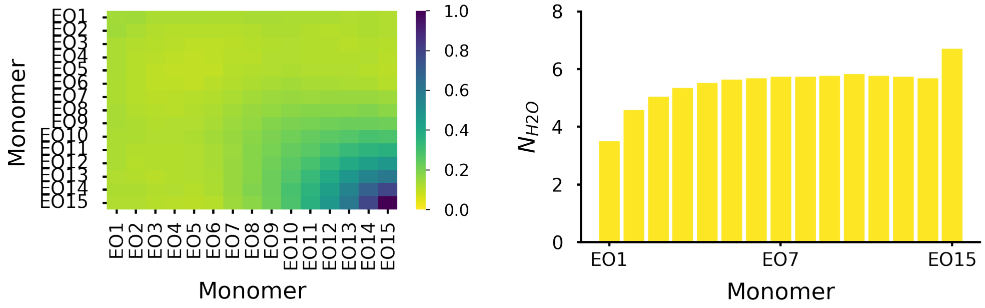

(a) EO-EO contacts (b) EO hydration

Figure S6: (a) Normalised intermolecular contact maps between the EO monomers and (b) normalised hydration of the oxygen atoms of the EO monomers for the EO-terminated polymer micelle.

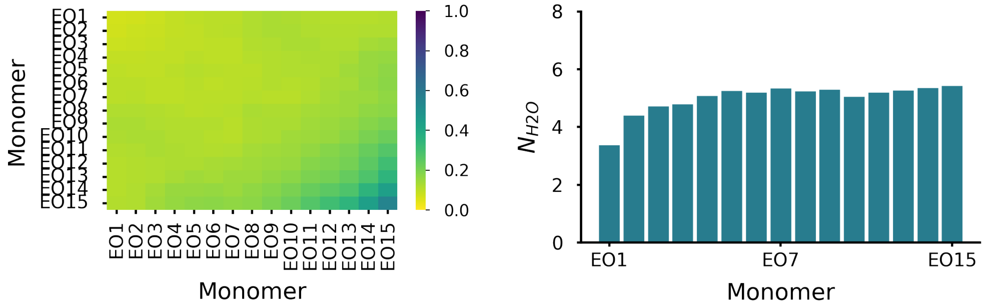

(a) EO-EO contacts (b) EO hydration

Figure S7: (a) Normalised intermolecular contact maps between the EO monomers and (b) normalised hydration of the oxygen atoms of the EO monomers for the ring polymer micelle.

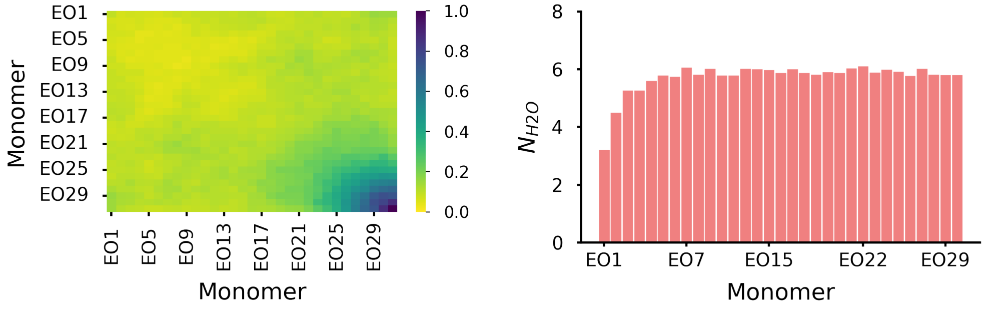

(a) EO-EO contacts (b) EO hydration

Figure S8: (a) Normalised intermolecular contact maps between the EO monomers and (b) normalised hydration of the oxygen atoms of the EO monomers for the diblock polymer micelle.

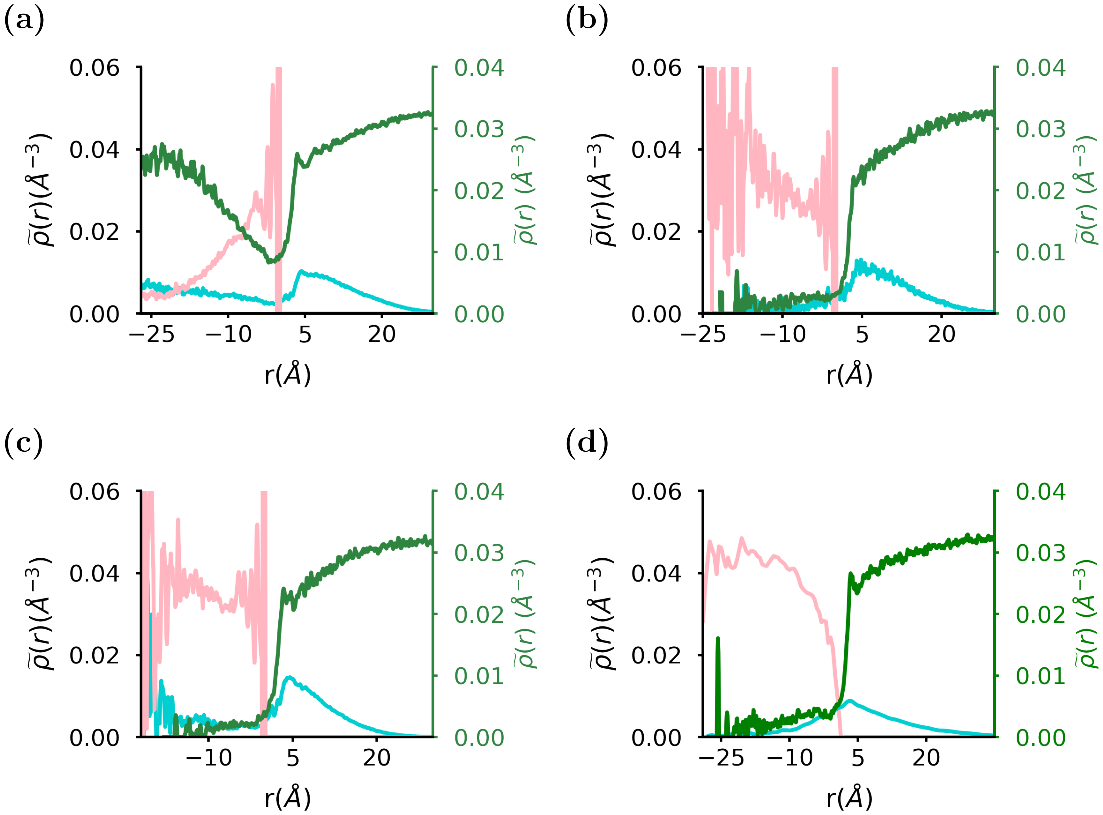

Figure S9: **Intrinsic density of micelle components.** Intrinsic density of the (a) MA-terminated polymer micelle, (b) EO-terminated polymer micelle and (c)ring polymer micelle. The intrinsic density of the MA monomers is displayed in pink, EO in blue and water in dark green.

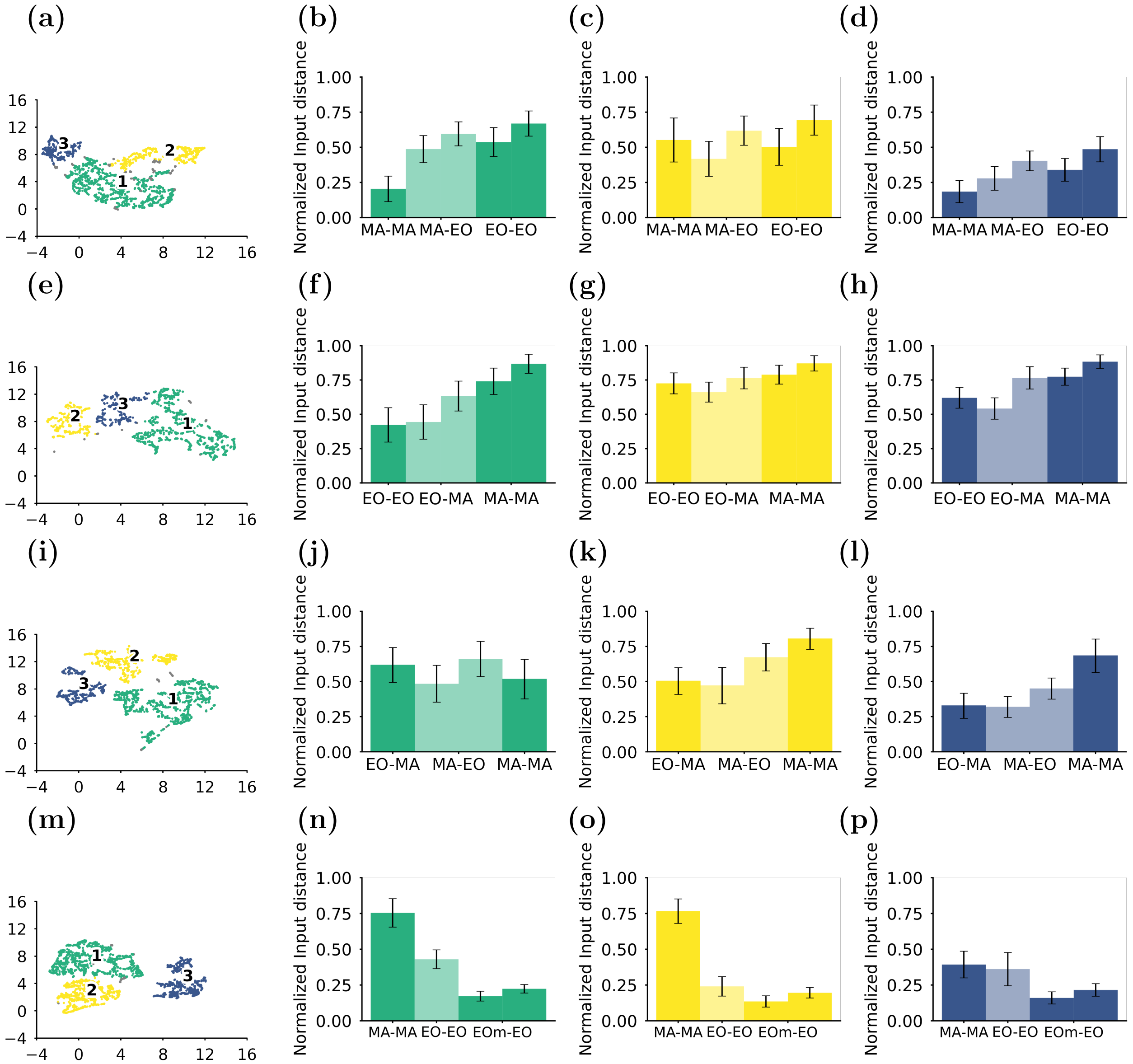

Figure S10: **UMAP embedded space and average cluster distances** UMAP embedded space clustered by HDBSCAN of (a) linear triblock MA-EO-MA polymers, (e) linear triblock EO-MA-EO polymers, (i) ring MA-EO polymers and (m) diblock polymer. Histograms of the average distances of each cluster. Cluster 1 for (b) linear triblock MA-EO-MA polymers, (f) linear triblock EO-MA-EO polymers, (j) ring MA-EO polymers and (j) diblock polymer. Cluster 2 for (c) linear triblock MA-EO-MA polymers, (g) linear triblock EO-MA-EO polymers, (k) ring MA-EO polymers and (o) diblock polymer. Cluster 3 (d) linear triblock MA-EO-MA polymers, (h) linear triblock EO-MA-EO polymers, (l) ring MA-EO polymers and (p) diblock polymer.

References

(1) Michaud-Agrawal, N.; Denning, E. J.; Woolf, T. B.; Beckstein, O. MDAnalysis: a toolkit for the analysis of molecular dynamics simulations. *J. Comput. Chem.* **2011**, *32*, 2319–2327.

(2) `https://pysoftwhere.readthedocs.io/en/latest/index.html`.

(3) Hagberg, A. A.; Schult, D. A.; Swart, P. J. Exploring Network Structure, Dynamics, and Function using NetworkX. Proceedings of the 7th Python in Science Conference. Pasadena, CA USA, 2008; pp 11 – 15.

(4) McInnes, L.; Healy, J.; Melville, J. Umap: Uniform manifold approximation and projection for dimension reduction. *arXiv preprint arXiv:1802.03426* **2018**,

(5) McInnes, L.; Healy, J.; Astels, S. hdbscan: Hierarchical density based clustering. *J. Open Source Softw.* **2017**, *2*, 205.

(6) Ziolek, R. M.; Smith, P.; Pink, D. L.; Dreiss, C. A.; Lorenz, C. D. Unsupervised learning unravels the structure of four-arm and linear block copolymer micelles. *Macromolecules* **2021**, *54*, 3755–3768.
